# Zinc regulates microglial polarization and inflammation through IKBα after spinal cord injury and promotes neuronal repair and motor function recovery in mice

**DOI:** 10.3389/fphar.2025.1510372

**Published:** 2025-01-29

**Authors:** Daoyong Li, Mingyu Bai, Zhanpeng Guo, Yang Cui, Xifan Mei, He Tian, Zhaoliang Shen

**Affiliations:** ^1^ The Third Affiliated Hospital of Jinzhou Medical University, Jinzhou City, Liaoning, China; ^2^ Key Laboratory of Liaoning Medical Organization Engineering, Jinzhou, Liaoning, China; ^3^ School of Basic Medicine, Jinzhou Medical University, Jinzhou, Liaoning, China

**Keywords:** spinal cord injury, zinc, microglial polarization, inflammatory response, functional recovery

## Abstract

**Introduction:**

Spinal cord injury (SCI) leads to severe inflammation and neuronal damage, resulting in permanent loss of motor and sensory functions. Zinc ions have shown potential in modulating inflammation and cellular survival, making them a promising therapeutic approach for SCI. This study investigates the mechanisms of zinc ion treatment in SCI, focusing on its effects on inflammation.

**Methods:**

We used transcriptomic analysis to identify key pathways and genes involved in the inflammatory response in a mouse model of SCI. *In vitro* studies assessed the impact of zinc ions on inflammation, cell polarization, and apoptosis. IKBα expression was evaluated as a potential target of zinc ions, both in cultured cells and *in vivo*.

**Results:**

Transcriptomic analysis revealed that zinc ions modulate inflammatory pathways through IKBα, which inhibits NF-κB activity. *In vitro*, zinc treatment upregulated IKBα expression, reducing inflammation, polarization, and apoptosis. These results were confirmed in the SCI mouse model, where zinc ions also reduced inflammation and cell death.

**Discussion:**

Our findings highlight a novel mechanism by which zinc ions regulate inflammation in SCI by upregulating IKBα and inhibiting NF-κB activation. This suggests potential therapeutic applications of zinc ions in SCI and other inflammatory conditions, warranting further investigation into their clinical benefits.

## 1 Introduction

SCI is a catastrophic neurological disorder characterized by direct physical damage to the spinal cord and a subsequent complex cascade of secondary injuries (Huang et al., 2022). These secondary responses, including inflammation, oxidative stress, apoptosis, and glial scar formation, exacerbate the already compromised neural tissue, leading to further loss of neurological function (Pajarillo et al., 2022). SCI patients often face long-term disability, with severe motor and sensory impairments significantly affecting their quality of life and social participation (Iaffaldano et al., 2021).

Inflammatory responses play a central role in the pathophysiology of SCI. Following mechanical injury to the spinal cord, an initial primary injury is immediately triggered, after which inflammatory responses are rapidly activated, becoming a major driver of secondary damage (Brennan et al., 2022). This process involves multiple cellular and molecular mechanisms, where microglia at the injury site and peripherally infiltrating macrophages rapidly adopt a pro-inflammatory phenotype, releasing copious amounts of inflammatory mediators such as tumor necrosis factor-α (TNF-α), interleukin-1β (IL-1β), and interleukin-6 (IL-6) (Fan et al., 2020; Van Broeckhoven et al., 2022). These mediators not only exacerbate the local inflammatory environment but also activate downstream signaling pathways, such as nuclear factor κB (NF-κB) and mitogen-activated protein kinase pathways, further amplifying the inflammatory response (Muñoz García et al., 2024). Additionally, inflammatory mediators induce a cascade of cytokine reactions, leading to the recruitment of more immune cells to the injury site, creating a vicious cycle that persistently aggravates neural tissue damage (Groeger et al., 2010). Therefore, inflammatory responses present both challenges and opportunities in the neuroprotection and repair processes following SCI. A thorough understanding and effective regulation of these responses are crucial for mitigating the long-term impact of SCI.

Microglia are the primary immune cells in the central nervous system, and they swiftly activate and exhibit different polarization states following SCI, primarily classified into M1 and M2 types (Caffarel and Braza, 2022; Mizobuchi and Soma, 2021). M1 microglia are pro-inflammatory, secreting large amounts of inflammatory mediators such as TNF-α, IL-1β, and nitric oxide, which exacerbate neuronal damage and death, thereby expanding the injury zone (Zhao et al., 2020). In contrast, M2 microglia possess anti-inflammatory and reparative properties, secreting anti-inflammatory factors like IL-10 and TGF-β, promoting neuroprotection and tissue repair (Tan et al., 2019). However, the post-SCI environment typically favors M1 polarization, leading to sustained inflammation and neuronal damage. Therefore, regulating microglial polarization to promote a shift towards the M2 phenotype is a crucial strategy for reducing inflammation and facilitating neural repair (Liu et al., 2020; Sun et al., 2022).

Zinc ions, as a crucial trace element, perform various biological functions in the nervous system, including neurotransmitter release, synaptic plasticity, neuroprotection, and injury repair. Dysregulation of zinc homeostasis is linked to the pathophysiology of neurodegenerative diseases such as Alzheimer’s, Parkinson’s, and ischemic stroke (Mo et al., 2022). Zinc promotes SCI recovery by accelerating neuronal autophagy and inhibiting apoptosis, and it regulates NLRP3 inflammasome activity via autophagy and ubiquitination. Additionally, zinc alleviates neuronal apoptosis by modulating mitochondrial quality control post-SCI (Jiang et al., 2023; Li et al., 2020).

Based on this background, our study aims to investigate whether zinc ions can modulate the inflammatory response of microglia by regulating key inflammatory genes, and whether this modulation impacts neuroprotection and motor function recovery post-SCI. This research will elucidate the mechanisms of zinc ions in SCI treatment, providing a scientific basis for developing new therapeutic strategies and ultimately improving the quality of life for SCI patients.

## 2 Materials and methods

### 2.1 Cell culture and treatment

Mouse BV2 microglia and PC12 neuron cell lines were purchased from the cell bank of the Chinese Academy of Sciences. They were cultured in Dulbecco’s modified Eagle’s medium (DMEM, Hyclone, UT, United States) supplemented with 10% fetal bovine serum (FBS, Gibco, CA, United States), 100 units/mL penicillin and 100 μg/mL streptomycin (Gibco, Grand Island, NY, United States). All cells were cultured in a humidified incubator containing 5% CO2 and 95% air at 37°C, and the cell culture medium was changed every 2 days. After the cell state is stabilized, the LPS (0.5 μg/mL) and BV2 cells were incubated for 24 h to induce the cells to be in a state of inflammatory damage, and then used in each group of experiments. Then, Zinc Gluconate (ZnG) (100 μMol) was incubated with the treated BV2 cells for 24 h. Then, the cell tissues of each group were collected for subsequent experiments.

### 2.2 Cell viability

BV2 cells were seeded in 96-well plates (5,000 cells/well) and incubated at 37°C with 5% CO2 for 24 h. Different doses of Zinc were then added to different wells and the cells were incubated for another 24. Finally, 20 μL CCK-8 (Btyotime, China) solution and 180 μL fresh medium were added to each well, and incubated together at 37°C for 1 h, and the absorbance measured at 450 nm using a microplate reader (Synergy-2, BioTek, Winooski, VT, United States).

### 2.3 Cell transfection

To silence NF-kappa-B inhibitor alpha (IKBα), si-IKBα (genefarma, Shanghai, China) was transfected into BV2 cells in the presence of Lipofectamine 3,000 reagent (Thermo Scientific, United States) according to the manufacturer’s protocol. At the same time, a negative control (NC) was used for simultaneous transfection. Use Western blotting (WB) to confirm transfection efficiency. IKBα siRNA, sense: 5′-CCA​UGA​AGG​ACG​AGG​AGU​ATT-3′; anti-sense:5′-UACUCCUCGUCCUUCAUGGTT-3′. NC siRNA, sense: 5′-UUC​UCC​GAA​CGU​GUC​ACG​UTT -3′; anti-sense: 5′-ACG​UGA​CAC​GUU​CGG​AGA ATT -3′.

### 2.4 Quantitative real-time PCR

Total RNA was extracted using Trizol (Qiagen, Hilden, Germany) from BV2 cells, PC12 cells, or 1.5 cm long spinal cord tissue centered at the site of spinal cord injury, according to the manufacturer’s protocol (Sang et al., 2024). RNA was then quantified using the NanoDrop 1,000 spectrophotometer (Thermo Scientific, United States), followed by reverse transcription using the Superscript^®^ III reverse transcription Kit (Invitrogen, Waltham, Ma, United States). Quantitative real-time PCR (qRT-PCR) was performed using the Quantifast SYBR^®^ green PCR kit (Qiagen, Hilden, Germany) on the Applied Biosystems 7500HT Fast Real-Time PCR System. Murine ribosomal protein S18 (RPS18) was then amplified on all samples as a housekeeping gene and internal control to explain changes in mRNA levels. Finally, the (1 + e)−ΔΔCT method was used to compare the target gene in the experimental group with the corresponding target gene in the control group. All primer sequences used in this experiment are shown in Table 1.

**TABLE 1:** Primers used for qRT-PCR.

Gene	Forward	Reverse
IKBα	GCT​GGA​AGG​CAG​AAG​TGA​AGG	TGC​AGG​CTC​TAT​CGG​GTA​TTT
MAP2	GAC​AGA​GAA​ACA​GCA​GAG​GAA​GTG	TGT​TCT​GAT​GCT​GGC​GAT​GGT
IL-1β	GTT​GAC​GGA​CCC​CAA​AAG​AT	AAG​GTC​CAC​GGG​AAA​GAC​AC
IL-6	TAG​TCC​TTC​CTA​CCC​CAA​TTT​CC	TTG​GTC​CTT​AGC​CAC​TCC​TTC
CD206	AGT​GAT​GGT​TCT​CCT​GTT​TCC	GGT​GTA​GGC​TCG​GGT​AGT​AGT
CD68	ACG​TAT​TGG​AAG​GAG​ATT​ACA​GCT	TCT​GTC​AGC​GTT​ACT​ATC​CCG​C
Arg-1	GAA​CAC​GGC​AGT​GGC​TTT​AAC	TGC​TTA​GCT​CTG​TCT​GCT​TTG​C
GAPDH	CAA​GTT​CAA​CGG​CAC​AGT​CAA​G	ACA​TAC​TCA​GCA​CCA​GCA​TCA​C
RPS18	AGG​ATG​TGA​AGG​ATG​GGA​AG	TTC​TTC​AGC​CTC​TCC​AGG​TC

### 2.5 Neuron-microglia cocultures

As shown in Figure 6A, Transwell establishes a PC12 cell-BV2 cell system that is independent of contact, as described previously. Briefly, PC12 cells (8 × 10^5^/well) were first seeded in 6-well plates and treated with LPS (0.5 μg/mL) for 24 h. At the same time, the BV2 cells (4 × 10^5^/well) inoculated in the inserts of each group were incubated together for 24 h under different treatment factors. Then, the insert washed with PBS was directly added above the PC12 cells, and the culture was continued in a 6-well plate. The 2 cells were cultured together for 12 h, sharing the same medium. After the end of co-cultivation, neurotoxicity was analyzed using lactate dehydrogenase (LDH) release and qPCR on MAP2 gene expression.

### 2.6 Determination of LDH content

After the PC12 cells in each group were treated with different reagent combinations, the activity of LDH was measured using a test kit (Jiancheng Bioengineering Research Institute, China) according to the manufacturer’s protocol. The absorbance was then measured at 450 μm using a Varioskan Flash Reader (Thermo Scientific, Waltham, MA, United States).

### 2.7 Animals

According to previous studies (Xu et al., 2023), animal experiments were conducted in C57BL/6 mice (20–35 g, 10–12 weeks) each half of females and males. All mice were purchased from Beijing Vital River Laboratory Animal Technology Co., Limited in Beijing, China. All operative procedures were approved by the animal experimental ethics committee of Jinzhou Medical University. All animals were kept under controlled conditions in a 12 h light/dark cycle at 23°C, with food and water available *ad libitum*.

### 2.8 SCI model and animal grouping

The model of contusion spinal cord injury in mice was established using the modified weight-drop method. Mice were intraperitoneally injected with ketamine (75 mg/kg) and xylazine (3 mg/kg) for anesthesia. The hair centered on T9 was cut and the skin disinfected, then the skin was cut, the muscle tissue separated, and the spinal cord exposed. An impactor with a diameter of 2 mm and a weight of 12.5 g was used to strike the surface of the spinal cord from a height of 5 cm at T9, causing moderate contusion. Mice in a sham operation group underwent laminectomy but were not struck with an impactor. The wounds were then closed and the mice returned to the cage. They were helped to urinate twice a day until the voluntary urination function was restored. For the experimental procedure, the mice were categorized into four distinct groups: Sham (Laminectomy Only), SCI (Spinal Cord Injury), Zinc (Intraperitoneal ZnG (30 mg/kg) injection 2 h post-spinal cord injury surgery, 1 time/d until day 3.) and BAY 11–7,082 (Intraperitoneal ZnG (30 mg/kg) and injection 2 h post-spinal cord injury surgery, 1 time/d until day 3. And 10 μg/day BAY 11–7,082, injection 2 h post-spinal cord injury surgery, 1 time/d until day 3.)The timing and dose of ZnG and BAY 11–7,082 were based on previous studies (Deng et al., 2023).

### 2.9 Western blot analysis

BV2 cells were collected after 24 h of drug treatment. Mouse spinal cord tissue was collected on the third day after injury for protein analysis, and Western blotting was performed following Methods detailed in (Irrera et al., 2017). The main antibodies and concentration used in this experiment are as follows: anti-IL-1β (1:1,000, abcam, Cambridge, United Kingdom); anti-IL-6 (1:1,000, abcam, Cambridge, United Kingdom); anti-TNF-α (1:1,000, abcam, Cambridge, United Kingdom); anti-β-Actin (1:10,000, abcam, Cambridge, United Kingdom); anti-IKBα (1:1,000, abcam, Cambridge, United Kingdom); anti-CD68 (1:1,000, abcam, Cambridge, United Kingdom); anti-INOS (1:1,000, Cell Signaling Technology, Inc., Danvers, MA, United States); anti-Arg1 (1:1,000, abcam, Cambridge, United Kingdom); anti-CD206 (1:1,000, Cell Signaling Technology, Inc., Danvers, MA, United States); anti-MCP1 (1:500, abcam, Cambridge, United Kingdom). Finally, enhanced chemiluminescence reagents (Thermo Fisher Scientific) were used to visualize the results and ImageJ software (NIH, Bethesda, MD, United States) was used to analyze the density of protein bands.

### 2.10 Immunofluorescence analysis

Spinal cord slices from each experimental group were removed from storage at −80°C and rewarmed at room temperature for 2 h, and the cells from each experimental group were fixed with 4% paraformaldehyde in a cell culture dish. The slices and cells were then separately incubated with 0.3% tritonx-100 for 15 min and with goat serum for 2 h. Primary antibodies were then incubated with spinal cord slices and cells separately overnight in a 4°C humidified chamber. The main antibodies and concentration are as follows: anti-IL-1β (1:500 abcam, Cambridge, United Kingdom); anti-INOS (1:500 abcam, Cambridge, United Kingdom); anti-IL-6 (1:500 abcam, Cambridge, United Kingdom); anti-Arg-1 (1:500 abcam, Cambridge, United Kingdom); anti-Anti-β-III Tubulin (1:500, abcam, Cambridge, United Kingdom); anti-Iba-1 (1:1,000, abcam, Cambridge, United Kingdom); anti-α-Tubulin (1:1,000, abcam, Cambridge, United Kingdom); anti-GFAP (1:500, abcam, Cambridge, United Kingdom). The primary antibody was then eluted with PBS and the spinal cord tissue and cells were incubated with Alexa fluor 488 Goat anti rabbit IgG or Alexa fluor 594 Goat anti mouse IgG (1:250, Thermo Fisher Science) for 2 h at room temperature. The spinal cord tissue and cells were washed three times with PBS for 5 minutes and the nuclei stained with 40,6-diaminodinitro-2-benzoindole solution (DAPI) (Invitrogen, Carlsbad, CA, United States) for 20 min. Finally, results were observed under a fluorescence microscope (Olympus Hamburg, Germany), and the optical density of fluorescence was analyzed using ImageJ2x software.

### 2.11 TUNEL staining

TUNEL staining was performed on the spinal cord sections one days after SCI with an in situ apoptosis detection kit (Jiancheng Institute of Bioengineering, Nanjing, China). Spinal cord slices from each experimental group were rewarmed at room temperature for 2 h and then washed three times with PBS for 5 min each time. The washed spinal cord sections were cultured with 0.3% Triton X-100 at 4°C for 15 min and then washed again three times with PBS and incubated with TUNEL reaction mixture in a dark, humid environment at 37°C for 60 min. They were then washed again three times with PBS for 3 min each time, and incubated with DAPI for 10–15 min. Finally, the spinal cord sections were observed and photographed under a microscope.

### 2.12 Measurements of mitochondrial ROS

Mitochondrial ROS levels were quantified using MitoSOX™ Red fluorescent dye (M36008, Invitrogen) according to the manufacturer’s instructions. Cells were incubated in MitoSOX™ Red-containing medium for 30 min, washed, and then analyzed by flow cytometry. Data were analyzed using FlowJo v10.8.1 software.

### 2.13 Mitochondrial membrane potential assay (ΔψM)

Mitochondrial membrane potential was assessed using a JC-1 Mitochondrial Membrane Potential Assay Kit (Beyotime, China). Cells were stained with 2.0 μg/mL JC-1 dye at 37°C for 20 min, washed, and examined under a fluorescence microscope. The analysis involved the relative fluorescence ratio technique, comparing the red/green fluorescence ratios of apoptotic, necrotic, and viable cells. In healthy cells, JC-1 accumulates in the mitochondrial matrix, emitting red fluorescence, while a decrease in membrane potential results in monomeric JC-1 and green fluorescence. To maintain objectivity, an independent researcher captured five random images per group using a Leica DMi8 fluorescence microscope (Wetzlar, Germany). Fluorescence intensity per pixel was quantified using ImageJ software, with uniform threshold adjustments, and the average pixel intensity was calculated to represent the mean fluorescence intensity for each group.

### 2.14 Statistical analysis

SPSS statistical software (Chicago, Illinois, United States) was used for analysis. All data were expressed as mean ± standard deviation (SD). Shapiro-Wilk test was used to evaluate the data distribution. The two groups were analyzed by Mann-Whitney U test. When there were more than two groups, we used one-way ANOVA, Bonferroni’s post test (when the variances were equal) and Kruskal–Wallis test (when the variances were not equal) to compare multiple groups. BMS score was analyzed using a two-way ANOVA with Tukey’s *post hoc* test. For all tests, Significance levels were defined as p < 0.05 (*), p < 0.01 (**), p < 0.001 (***).

## 3 Results

### 3.1 Zinc ion treatment for spinal cord injury shows gene enrichment in the inflammatory response

**FIGURE 1:**
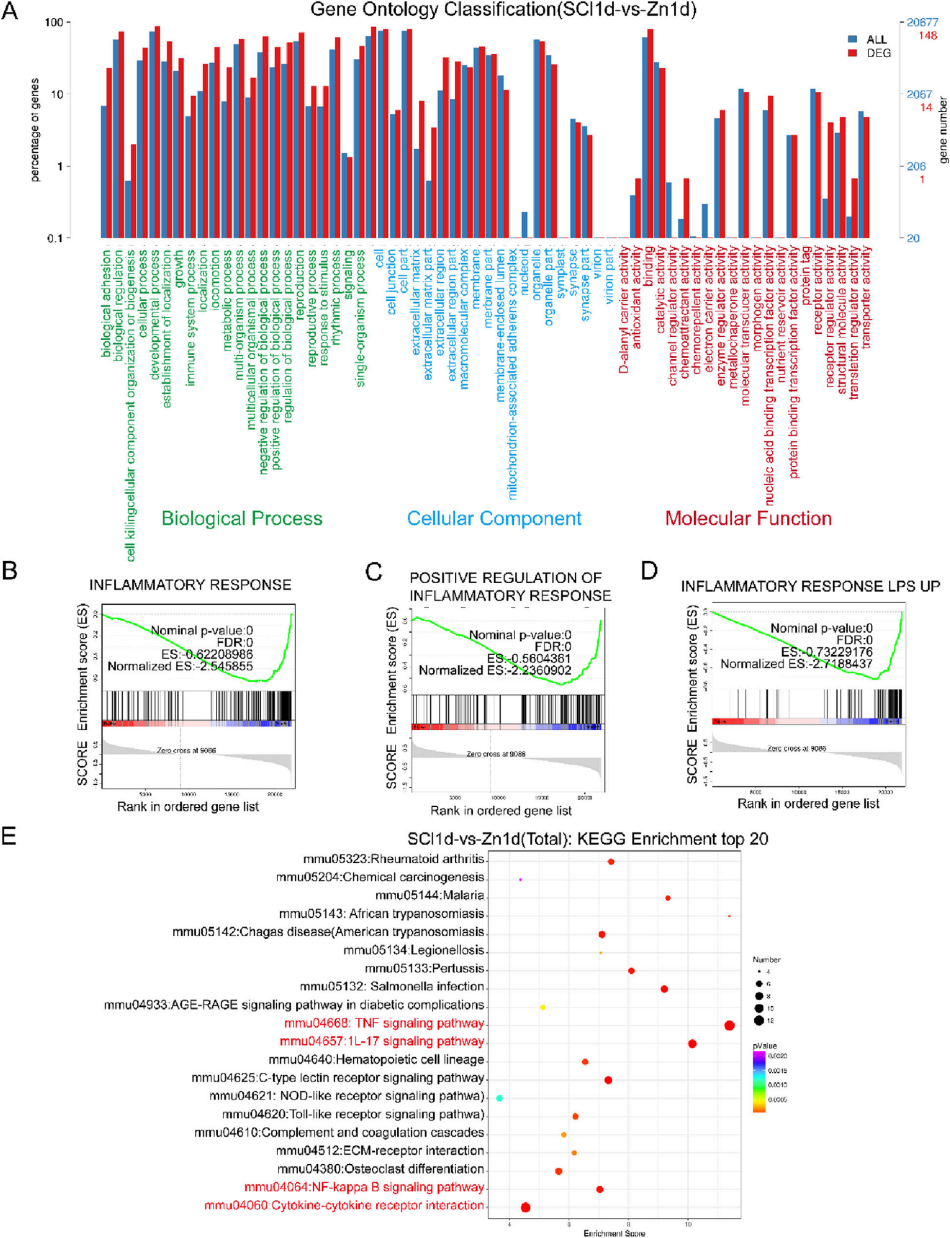
Zinc ion treatment for spinal cord injury shows gene enrichment in the inflammatory response. **(A)** Conduct GO enrichment analysis comparing SCI1d and Zinc1d groups. **(B–D)** Perform GSEA enrichment analysis comparing SCI1d and Zinc1d groups. **(E)** Execute KEGG enrichment analysis comparing SCI1d and Zinc1d groups.

One day post-SCI, upon zinc ion treatment, our GO enrichment analysis revealed that compared to the injured group, the zinc-treated group exhibited enriched biological processes related to factor regulation and signaling, indicating increased signaling protein transmission (Figure 1A). Subsequent GSEA enrichment analysis showed a significant number of genes enriched in gene sets associated with inflammatory response, positive regulation of the inflammatory response, and LPS-induced inflammation, highlighting the critical regulatory role of zinc ions in the inflammatory response of SCI mice (Figures 1B–D). Furthermore, our KEGG enrichment analysis identified changes in inflammatory pathways such as TNF signaling and NF-κB signaling (Figure 1E). These findings underscore the paramount importance of zinc ion’s regulatory effects on inflammation in SCI treatment.

### 3.2 Zinc ions inhibit the expression of inflammatory cytokines in BV2 cells

**FIGURE 2:**
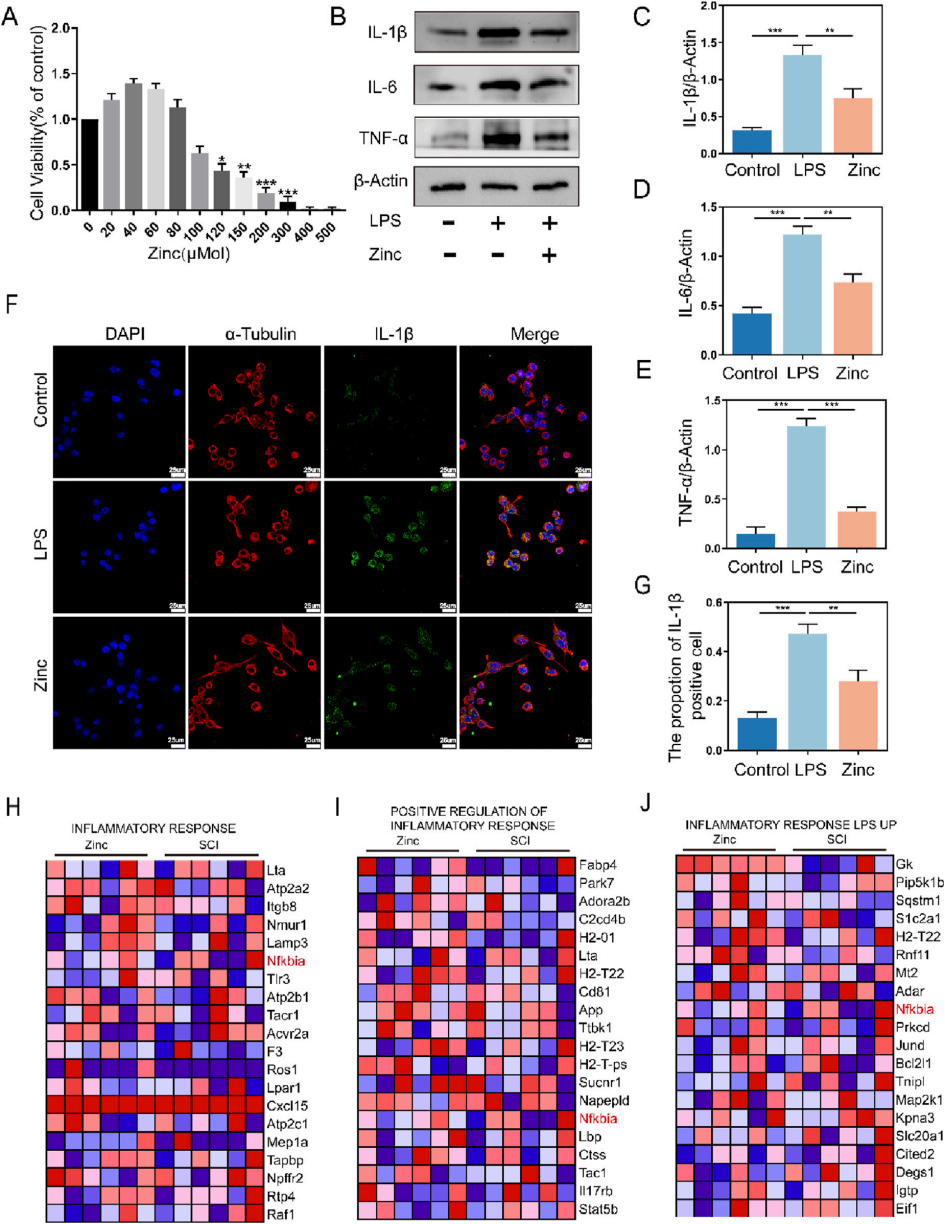
Zinc ions inhibit the expression of inflammatory cytokines in BV2 cells. **(A)** Assessment of BV2 cell viability under varying concentrations of Zinc (μMol). **(B–E)** Representative Western blot images and quantifications of IL-1β, IL6, and TNF-α in BV2 cells from Control, LPS (0.5 μg/mL), and Zinc (100 μMol) groups, n = 6. **(F, G)** Representative immunofluorescence images and quantifications of IL-1β in BV2 cells from Control, LPS, and Zinc groups, scale bar = 25 μm, n = 6. **(H–J)** Core genes identified through GSEA enrichment analysis comparing SCI1d and Zinc1d groups. Data presented the mean ± SD, *p < 0.05, **p < 0. 01, and ***p < 0. 001.

To validate the anti-inflammatory effects of zinc ions in vitro, we first determined the optimal concentration for zinc ions on cell viability (Figure 2A). Using Western blot analysis, we assessed the expression of inflammatory cytokines IL-1β, IL-6, and TNF-α, and found that zinc ions significantly inhibited their expression (Figures 2B–E). In the LPS-stimulated BV2 cell model, zinc ions effectively suppressed IL-1β expression (Figures 2F, G). After preliminarily confirming the anti-inflammatory effects of zinc ions, we further screened core genes from the GSEA-enriched gene sets and identified NFKbia as a pivotal gene consistently involved in inflammation clusters. As a transcriptional gene for IKBα, we hypothesize that zinc ions may alleviate inflammation post-SCI by promoting IKBα production.

### 3.3 Zinc ions elevate IκBα to suppress inflammatory cytokine expression in BV2 cells

**FIGURE 3:**
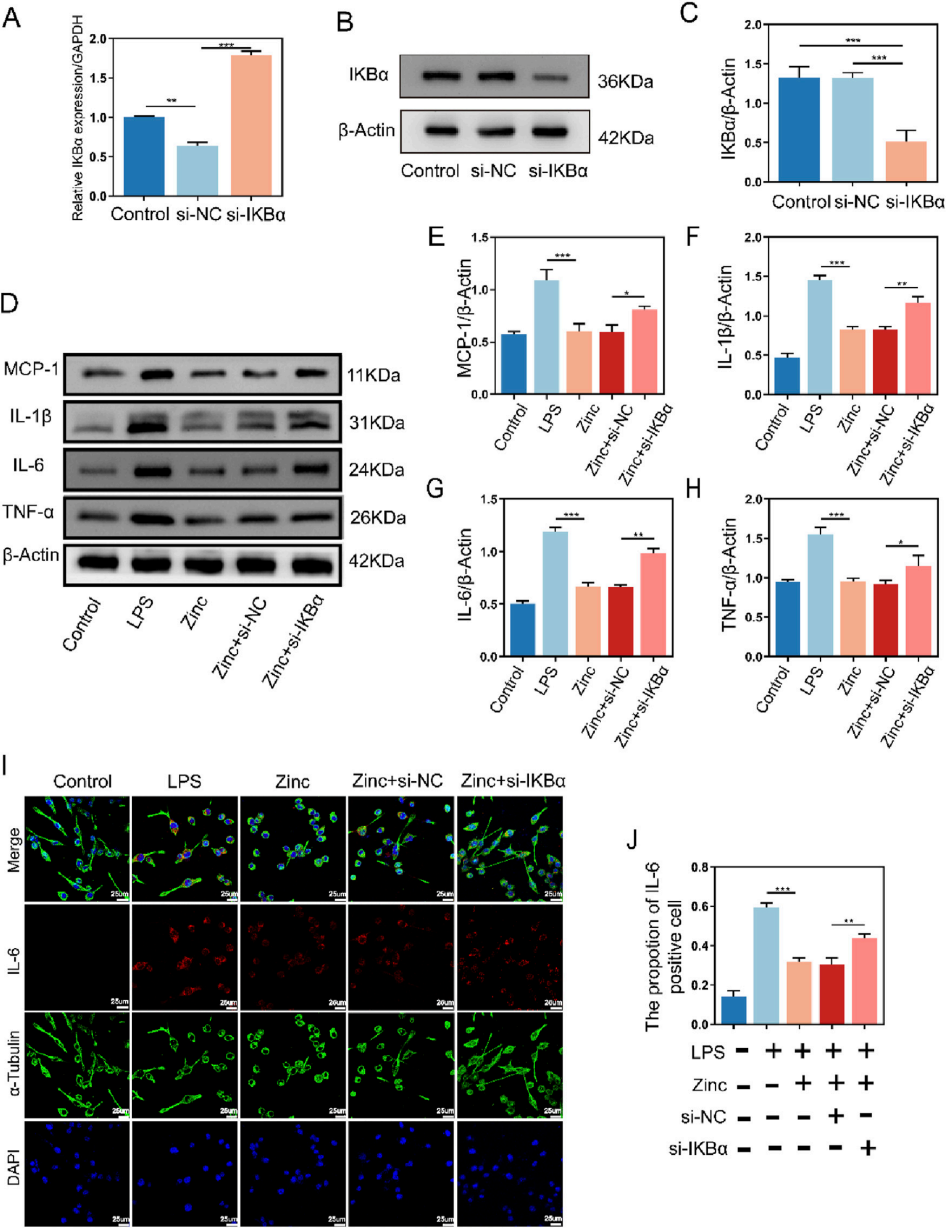
Zinc ions elevate IκBα to suppress inflammatory cytokine expression in BV2 cells. **(A)** Quantitative PCR analysis of IKBα expression in BV2 cells from Control, si-NC, and si-IKBα groups. **(B, C)** Representative Western blot images and quantifications of IKBα expression in BV2 cells from Control, si-NC, and si-IKBα groups, n = 6. **(D–H)** Representative Western blot images and quantifications of MCP1, IL-1β, IL6, and TNF-α expression in BV2 cells from Control, LPS (0.5 μg/mL), Zinc (100 μMol), Zinc + si-NC, and Zinc + si-IKBα groups, n = 6. **(I, J)** Representative immunofluorescence images and quantifications of IL6 in BV2 cells from Control, LPS (0.5 μg/mL), Zinc (100 μMol), Zinc + si-NC, and Zinc + si-IKBα groups, scale bar = 25 μm, n = 6. Data presented the mean ± SD, *p < 0.05, **p < 0. 01, and ***p < 0. 001.

To verify whether the increase in IKBα is crucial for the anti-inflammatory effects of zinc ions, we transfected BV2 cells to inhibit IKBα expression and confirmed the transfection efficiency (Figures 3A–C). Using Western blot analysis, we assessed the expression of MCP-1, IL-1β, IL-6, and TNF-α. Compared to the Zinc group, which showed inhibition of inflammatory cytokines, the Zinc + si-IKBα group lost this inhibitory effect, and the expression of inflammatory proteins increased. This indicates that the original inhibitory effect of zinc ions on inflammatory cytokine expression is counteracted by the inhibition of IKBα (Figures 3D–H). Cell fluorescence analysis of IL-6 confirmed the Western blot results, demonstrating that the increase in IKBα is closely related to the anti-inflammatory effects of zinc ions (Figures 3I, J).

### 3.4 Zinc ions elevate IκBα to promote M2 polarization while reducing M1 polarization in BV2 cells

**FIGURE 4:**
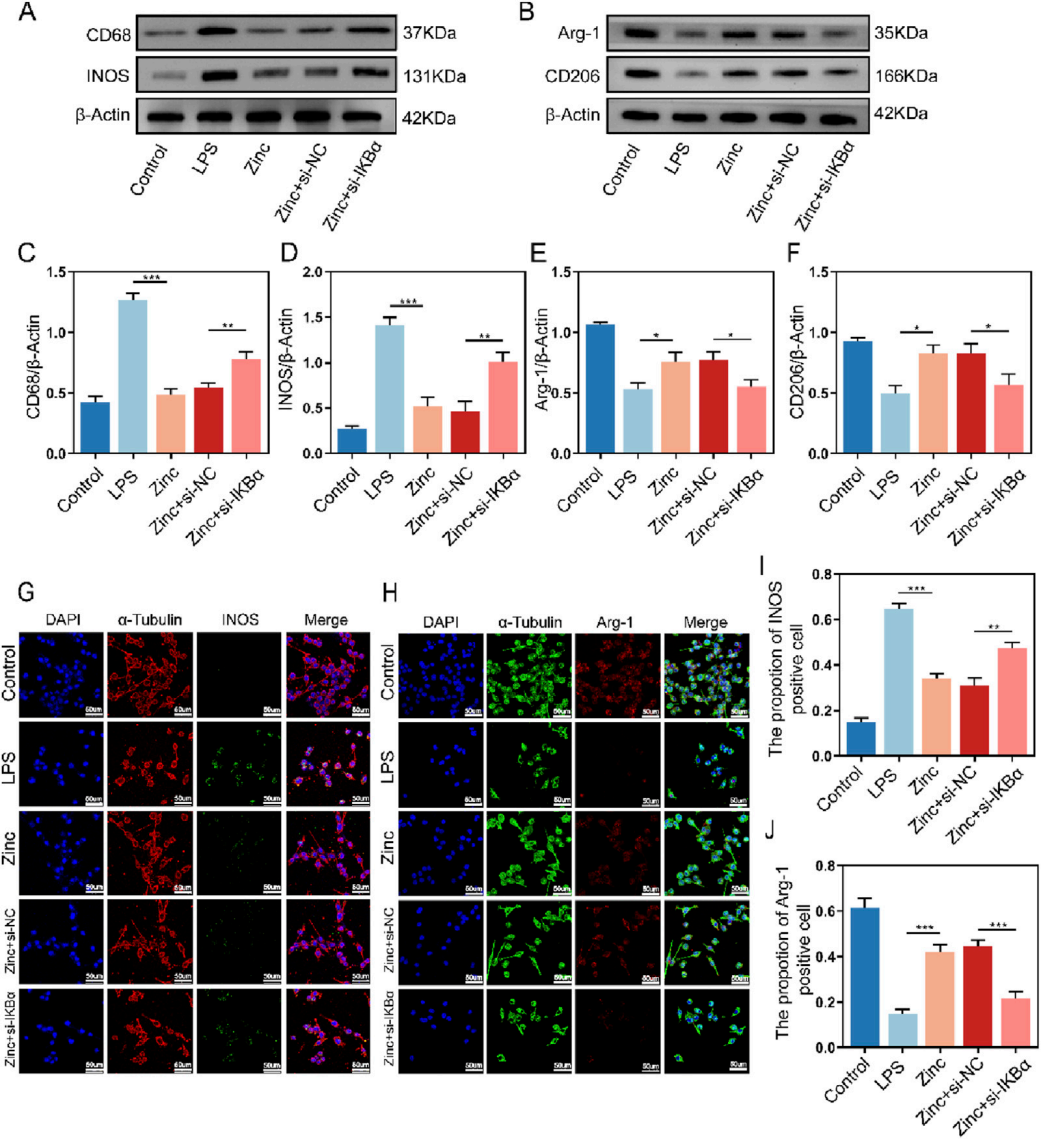
Zinc ions elevate IκBα to promote M2 polarization while reducing M1 polarization in BV2 cells. **(A, C, D)** Representative Western blot images and quantifications of CD68 and INOS expression in BV2 cells from Control, LPS (0.5 μg/mL), Zinc, Zinc + si-NC, and Zinc + si-IKBα groups, n = 6. **(B, E, F)** Representative Western blot images and quantifications of CD206 and Arg-1 expression in BV2 cells from Control, LPS (0.5 μg/mL), Zinc (100 μMol), Zinc + si-NC, and Zinc + si-IKBα groups, n = 6. **(G–J)** Representative immunofluorescence images and quantifications of INOS and Arg-1 in BV2 cells from Control, LPS, (0.5 μg/mL) Zinc, Zinc + si-NC, and Zinc + si-IKBα groups, scale bar = 50 μm, n = 6. Data presented the mean ± SD, *p < 0.05, **p < 0. 01, and ***p < 0. 001.

Microglia, as the crucial inflammatory cells in the central nervous system, have their polarization states closely tied to inflammatory responses. Through Western blot analysis, we found that under LPS stimulation, microglia exhibit increased expression of CD68 and INOS, indicating M1 polarization, which is associated with pro-inflammatory responses. Conversely, the expression of CD206 and Arg-1, markers of M2 microglia linked to anti-inflammatory effects, decreased. Zinc ion treatment shifted microglia towards the anti-inflammatory M2 polarization. However, when IKBα was knocked down, the ability of zinc ions to modulate microglial polarization was inhibited, causing microglia to re-polarize towards the pro-inflammatory M1 state (Figures 4A–F). Further cell fluorescence analysis confirmed that zinc ions promote microglial M2 polarization through upregulation of IKBα (Figures 4G–J).

### 3.5 Zinc ions elevate IκBα to inhibit oxidative stress in BV2 cells

**FIGURE 5:**
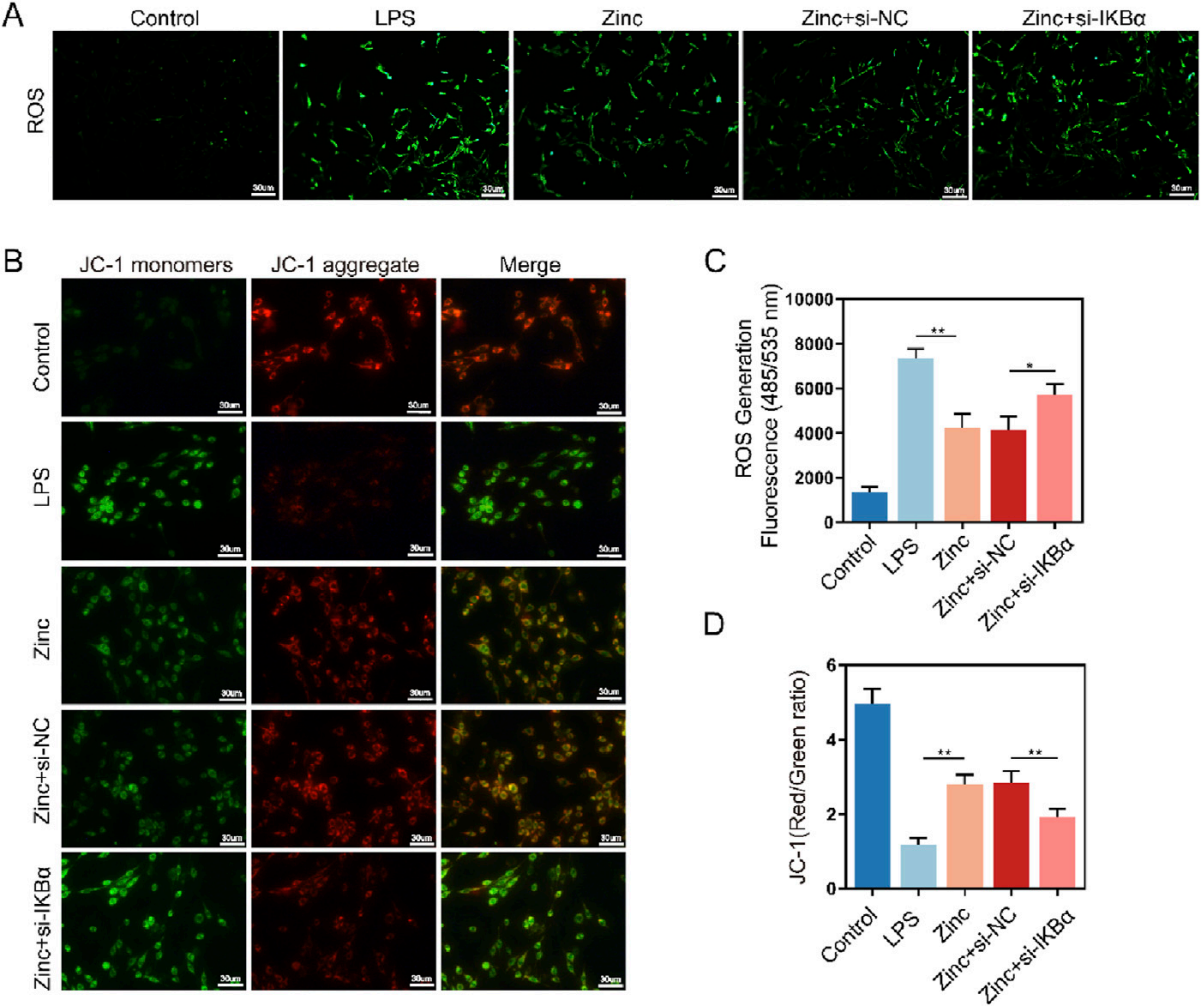
Zinc ions elevate IκBα to inhibit oxidative stress in BV2 cells. **(A, C)** Representative ROS fluorescence probe images and quantifications in BV2 cells from Control, LPS (0.5 μg/mL), Zinc (100 μMol), Zinc + si-NC, and Zinc + si-IKBα groups, scale bar = 20 μm, n = 6. **(B, D)** Representative JC-1 fluorescence probe images and quantifications in BV2 cells from Control, LPS (0.5 μg/mL), Zinc, Zinc + si-NC, and Zinc + si-IKBα groups, scale bar = 30 μm, n = 6. Data presented the mean ± SD, *p < 0.05, **p < 0. 01, and ***p < 0. 001.

Microglial inflammatory responses and polarization are closely linked to mitochondrial homeostasis. To further investigate the effect of zinc ions on mitochondria in microglia, we used ROS probes to detect intracellular ROS generation. Zinc ions significantly reduced ROS production; however, this inhibitory effect was abolished with the knockdown of IKBα (Figures 5A, C). Subsequently, using the JC-1 assay kit, we assessed mitochondrial membrane potential stability. We found that zinc ions depend on increased IKBα expression to maintain mitochondria predominantly in an aggregated state, thereby stabilizing the mitochondrial membrane potential (Figures 5B, D). Our experiments further confirmed the critical role of IKBα in zinc ion-mediated maintenance of intracellular homeostasis.

### 3.6 Zinc ions elevate IκBα to inhibit the pro-apoptotic effect of BV2 cells on PC12 cells

**FIGURE 6:**
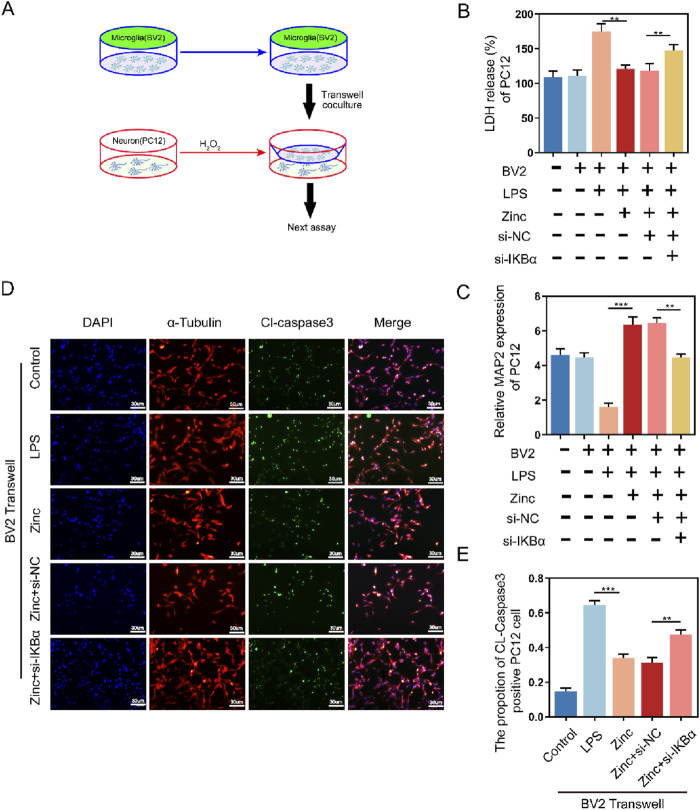
Zinc ions elevate IκBα to inhibit the pro-apoptotic effect of BV2 cells on PC12 cells. **(A)** Schematic illustration of Transwell assay with BV2 and PC12 cells. **(B)** LDH activity and quantifications in PC12 cells following Transwell exposure to Control, LPS (0.5 μg/mL), Zinc (100 μMol), Zinc + si-NC, and Zinc + si-IKBα groups, n = 6. **(C)** MAP-2 expression and quantifications in PC12 cells following Transwell exposure to Control, LPS (0.5 μg/mL), Zinc (100 μMol), Zinc + si-NC, and Zinc + si-IKBα groups, n = 6. **(D, E)** Representative immunofluorescence images and quantifications of CL-Caspase3 in PC12 cells following Transwell exposure to Control, LPS (0.5 μg/mL), Zinc (100 μMol), Zinc + si-NC, and Zinc + si-IKBα groups, scale bar = 30 μm, n = 6. Data presented the mean ± SD, *p < 0.05, **p < 0. 01, and ***p < 0. 001.

Microglia are also crucial for neuronal survival. To further explore the interaction between microglia and neurons, we used a Transwell system, culturing BV2 cells in the upper chamber and PC12 cells in the lower chamber (Figure 6A). Zinc-treated BV2 cells significantly enhanced the survival of PC12 cells, as evidenced by the LDH assay showing increased PC12 cell survival. Additionally, MAP2 expression analysis in PC12 cells revealed that zinc-treated PC12 cells exhibited more mature neuronal phenotypes. However, these protective effects of zinc ions were diminished when IKBα was knocked down in BV2 cells (Figures 6B, C). Cell fluorescence imaging further demonstrated that zinc-treated BV2 cells reduced CL-Caspase3 expression in PC12 cells, an effect that was inhibited with the knockdown of IKBα in BV2 cells (Figures 6D, E).

### 3.7 Zinc ions elevate IκBα to inhibit cytokine expression and M1 polarization of microglia in mice with spinal cord injury

**FIGURE 7:**
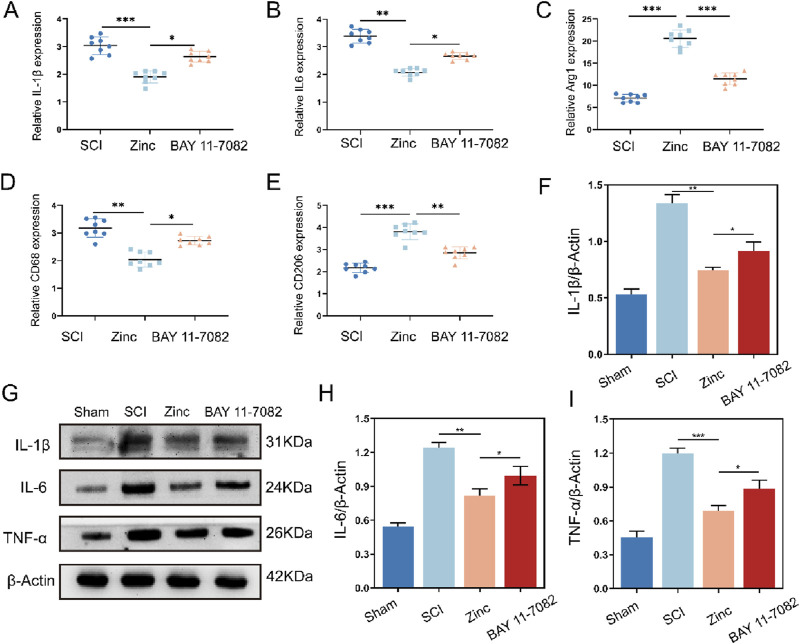
Zinc ions elevate IκBα to inhibit cytokine expression and M1 polarization of microglia in mice with spinal cord injury. **(A–E)** Quantitative PCR analysis of IL-1β, IL-6, Arg1, CD68, and CD206 in spinal cord tissues from SCI, Zinc (30 mg/kg), and BAY11-7,082 groups of mice, n = 8. **(F–I)** Representative Western blot images and quantifications of IL-1β, IL-6, and TNFα in spinal cord tissues from SCI, Zinc, and BAY11-7,082 groups of mice, n = 6. Data presented the mean ± SD, *p < 0.05, **p < 0. 01, and ***p < 0. 001.

In vitro experiments demonstrated that zinc ions play a positive role in reducing the expression of inflammatory factors, regulating microglial polarization, and protecting neurons, with these effects being closely associated with increased intracellular IKBα. To comprehensively validate our findings, we conducted further experiments in a mouse model of spinal cord injury. Using PCR, we measured the expression of IL-1β, IL-6, CD68, CD206, and Arg1 in the injured spinal cords. Zinc ions suppressed the transcription of pro-inflammatory cytokines and markers while enhancing the transcription of anti-inflammatory markers. However, these effects were blocked when the mice were treated with the IKBα inhibitor BAY 11–7,082 (Figures 7A–E). Western blot analysis confirmed these results at the protein level (Figures 7F–I). Additionally, tissue fluorescence analysis showed that zinc ions inhibited M1 polarization and promoted M2 polarization of IBA1-positive microglia in the injured spinal cords, an effect reversed by BAY 11–7,082 (Supplementary Figures 1A–D). These findings corroborate our in vitro results, indicating that zinc ions modulate microglial inflammation and polarization by upregulating IKBα.

### 3.8 Zinc ions elevate IκBα, enhancing motor function, reducing neuronal apoptosis, and limiting glial scar formation in mice with spinal cord injury

**FIGURE 8:**
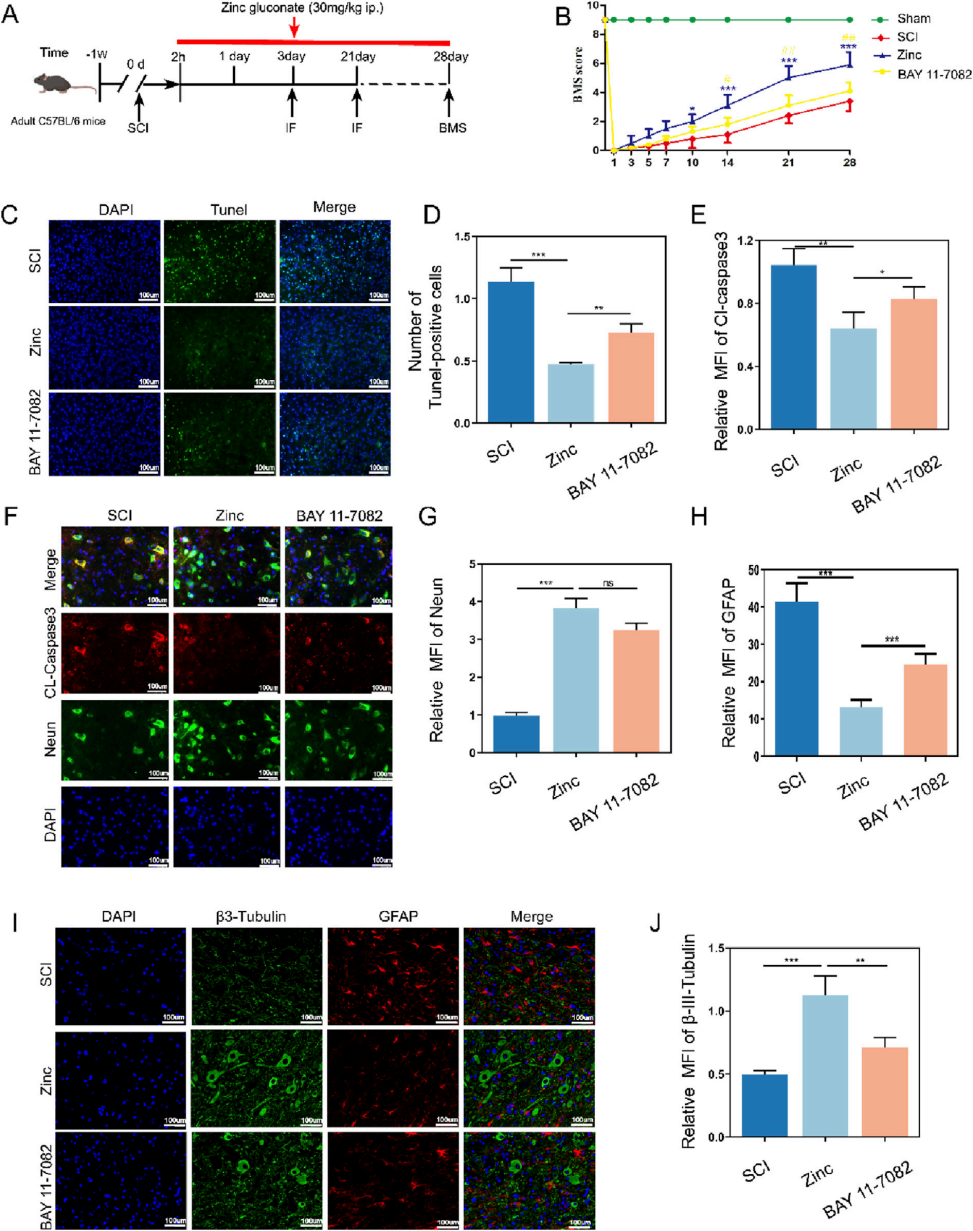
Zinc ions elevate IκBα, enhancing motor function, reducing neuronal apoptosis, and limiting glial scar formation in mice with spinal cord injury. **(A)** Schematic diagram of post-spinal cord injury detection in mice. **(B)** BMS scores in mice from SCI, Zinc, and BAY11-7082 groups. **(C, D)** Representative Tunel staining images and quantifications of spinal cord tissues from SCI, Zinc (30 mg/kg), and BAY11-7082 groups, scale bar = 100 μm, n = 6. **(E–G)** Representative immunofluorescence images and quantifications of CL-Caspase3 and Neun in spinal cord tissues from SCI, Zinc, and BAY11-7082 groups, scale bar = 100 μm, n = 6. **(H–J)** Representative immunofluorescence images and quantifications of GFAP and βIII-Tubulin in spinal cord tissues from SCI, Zinc, and BAY11-7082 groups, scale bar = 100 μm, n = 6. Data presented the mean ± SD, *p < 0.05, **p < 0. 01, and ***p < 0. 001.

Finally, we investigated the recovery of mice after zinc ion treatment (Figure 8A). Mice treated with zinc ions showed a gradual improvement in motor function scores (Figure 8B). TUNEL staining and Cl-Caspase3 tissue fluorescence staining indicated that zinc ion treatment provided significant anti-apoptotic effects in spinal cord injury models (Figures 8C–G). Additionally, long-term tissue analysis revealed that zinc ions inhibited glial scar formation, as shown by reduced GFAP and βIII-Tubulin expression, an effect dependent on increased IKBα expression (Figures 8H–J). In summary, our in vivo studies confirm that zinc ions promote motor function recovery and reduce glial scar formation after spinal cord injury, with these effects being dependent on the upregulation of IKBα.

## 4 Discussion

In this study, we explored the potential mechanisms and therapeutic effects of zinc ions in the treatment of spinal cord injury (SCI). Through transcriptomic analysis, we revealed that zinc ions mitigate the pathological processes of SCI by modulating the inflammatory response (Ge et al., 2021). Our findings suggest that the anti-inflammatory effects of zinc ions may be mediated by upregulating IKBα expression. As a key regulator of inflammation, IKBα was shown in vitro to reduce the production of inflammatory factors, influence microglial polarization, and inhibit apoptosis in PC12 cells, with these effects being dependent on increased IKBα expression. Our in vivo experiments in a mouse model of spinal cord injury further confirmed these conclusions, supporting that zinc ions exert their anti-inflammatory effects and promote motor function recovery by upregulating IKBα.

SCI is a severe neurological condition characterized by an initial mechanical insult followed by a cascade of complex secondary reactions, with inflammation playing a central role (Tang et al., 2023; Vishwakarma et al., 2018). Post-SCI, the disruption of the blood-brain barrier and acute neuronal death lead to the release of numerous intracellular and extracellular molecules (Man Hoi and Yue, 2023). These molecules, acting as damage-associated molecular patterns (DAMPs), trigger local immune responses (Bortolotti et al., 2018). The initiation of inflammation involves multiple cell types, including microglia, astrocytes, and infiltrating peripheral immune cells (Okazaki et al., 2016). These cells collectively release a series of inflammatory mediators, such as TNF-α, IL-1β, and IL-6. The expression of these cytokines rapidly increases following SCI, directly affecting neurons and glial cells, causing cell death and dysfunction (Mo et al., 2021). Additionally, they influence the polarization state of microglia, further amplifying the inflammatory response. Microglia, the intrinsic immune cells of the central nervous system, transition from a resting state to an activated state post-SCI, predominantly polarizing towards the M1 phenotype (Han et al., 2018; Liu et al., 2023). M1 microglia exhibit a pro-inflammatory profile, secreting large amounts of inflammatory cytokines, exacerbating local inflammation and neuronal damage (Lam et al., 2017). Over time, microglia can shift towards the M2 phenotype, which exhibits anti-inflammatory and reparative properties, secreting anti-inflammatory cytokines such as IL-10 and transforming growth factor-β (TGF-β), thereby promoting tissue repair and neuroregeneration (Ghosh et al., 2016). Current research highlights the importance of regulating microglial polarization and balancing the expression of pro-inflammatory and anti-inflammatory cytokines to mitigate secondary damage and promote functional recovery after SCI (Jiang et al., 2018; Liu et al., 2016).

IκBα (Inhibitor of κB α) is a critical inhibitor of the nuclear factor-κB (NF-κB), playing a significant role in the inflammatory response0. NF-κB is a family of transcription factors involved in regulating the expression of various inflammation-related genes. In a resting state, NF-κB is bound to IκBα in the cytoplasm, keeping it inactive (Colleran et al., 2011; Mamun et al., 2024). However, following SCI, injury signals activate the NF-κB pathway through various mechanisms, leading to the phosphorylation and degradation of IκBα (Zhang et al., 2021). This process releases NF-κB, allowing it to translocate to the nucleus and initiate the transcription of inflammation-related genes (Lv et al., 2021). The dysregulation of IκBα is central to the inflammatory response post-SCI. Studies indicate that excessive degradation of IκBα results in sustained activation of NF-κB, which in turn leads to the overexpression of inflammatory mediators, exacerbating neuronal damage and inflammation. Thus, modulating the stability and function of IκBα and balancing the activity of the NF-κB pathway are crucial for mitigating secondary damage and promoting functional recovery after SCI. Current research suggests that pharmacological interventions or gene therapy targeting IκBα expression and function could offer new therapeutic strategies for SCI.

In the inflammatory response following SCI, zinc ions play a multifaceted and crucial role. Zinc is not only an essential trace element in the central nervous system but also involved in a wide range of biological processes, including enzymatic catalysis, protein structure stabilization, and intracellular signal transduction (Wang et al., 2023). Extensive research has uncovered zinc intricate regulatory functions within cells, such as mitigating neuronal apoptosis by maintaining mitochondrial quality control, thereby protecting nerve cells from further damage (Bai et al., 2024). Additionally, zinc ions have shown potential in inhibiting certain inflammatory signaling pathways that could lead to pyroptosis post-SCI (Ye et al., 2022). Our research team has provided the first experimental evidence demonstrating that zinc ions alleviate the inflammatory response by upregulating IκBα (Inhibitor of κB α) expression. This finding is significant for understanding the mechanisms of zinc ions in SCI.

## 5 Conclusion

In summary, our comprehensive transcriptomic analysis has unveiled a novel mechanism of zinc ions in the treatment of spinal cord injury (SCI), particularly in regulating the inflammatory response. We discovered that zinc ions can reduce the production of inflammatory mediators and alleviate inflammation and cellular damage by upregulating IκBα expression. Consistent validation results from in vitro experiments and mouse models of SCI demonstrate that zinc ions effectively mitigate the inflammatory response and cellular apoptosis post-SCI, offering new perspectives for SCI treatment. Our research not only elucidates the potential mechanisms of zinc ions in SCI therapy but also provides a theoretical foundation for developing new therapeutic strategies. Future studies could further explore the interactions of zinc ions with other signaling pathways and the specific regulatory mechanisms of zinc ions at different stages of SCI, offering more precise and effective treatment approaches and targets for SCI.

## Data Availability

The original contributions presented in the study are included in the article/Supplementary Material, further inquiries can be directed to the corresponding authors.

## Abbreviations

SCI, Spinal cord injury; TNF, α tumor necrosis factor-α; IL, 1β interleukin-1β; IL, 6 interleukin-6; NF, κB nuclear factor κB.
