# Supplementary material for: Zinc regulates microglial polarization and inflammation through IKBα after spinal cord injury and promotes neuronal repair and motor function recovery in mice
Source: Front Pharmacol. 2025 Jan 29;16:1510372. doi: 10.3389/fphar.2025.1510372 (PMC11813752; doi:10.3389/fphar.2025.1510372)
Supplement: Supplementary file 1 [file DataSheet1.docx]

Fig2



IL-1β

IL6

TNF-α



β-actin

Fig3



IKBα

IL-1β



IL-6

MCP-5



TNF-α

β-Actin



β-Actin

Fig4



ARG1

CD68



CD206

INOS



β-Actin

β-Actin

Fig7



IL-1β

IL-6



TNF-α

β-Actin
