# Supplementary material for: Zinc regulates microglial polarization and inflammation through IKBα after spinal cord injury and promotes neuronal repair and motor function recovery in mice
Source: Front Pharmacol. 2025 Jan 29;16:1510372. doi: 10.3389/fphar.2025.1510372 (PMC11813752; doi:10.3389/fphar.2025.1510372)
Supplement: Supplementary file 2 [file Image1.pdf]

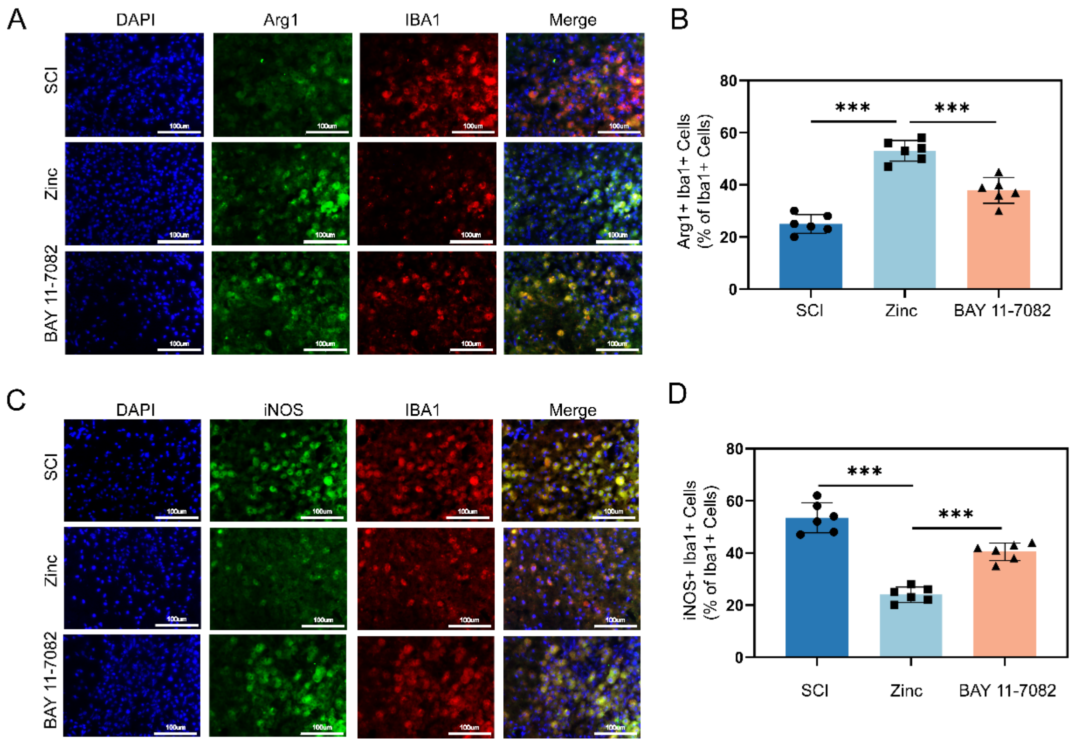


Supplementary Fig. 1. Zinc ions promote M2 polarization of microglia in the spinal cord of spinal cord injury mice by upregulating IKBα. (A, B) Representative immunofluorescence images and quantification of Arg1 in spinal cord tissues from SCI, Zinc, and BAY11-7082 groups, scale bar = 100 μm, n=6. (C, D) Representative immunofluorescence images and quantification of iNOS in spinal cord tissues from SCI, Zinc, and BAY11-7082 groups, scale bar = 100 μm, n=6. Data presented the mean ± SD, *p < 0.05, **p < 0. 01, and ***p < 0. 001.
